# Intrainstitutional Changes of the Treatment of Supracondylar Humerus Fracture in Children over a Period of 9 Years

**DOI:** 10.3390/children11010027

**Published:** 2023-12-26

**Authors:** Ferdinand Wagner, Amalia Boeriu, Pascal Eberz, Annabelle Weigert, Boris Michael Holzapfel, Wolfgang Böcker, Jochen Hubertus, Oliver Muensterer, Florian Bergmann, Christian Max Ziegler

**Affiliations:** 1 Department of Orthopaedics and Trauma Surgery, Musculoskeletal University Center Munich (MUM), LMU University Hospital, Ludwig-Maximilians-Universität München, Marchioninistrasse 15, 81377 Munich, Germanyboris.holzapfel@med.uni-muenchen.de (B.M.H.); christian.ziegler@med.uni-muenchen.de (C.M.Z.); 2 Institute of Health and Biomedical Innovation, Queensland University of Technology (QUT), Brisbane, QLD 4059, Australia; 3 Department of Pediatric Surgery, Dr. von Hauner Children’s Hospital, Ludwig-Maximilians-Universität München, Lindwurmstrasse 4, 80337 Munich, Germany; jochen.hubertus@elisabethgruppe.de (J.H.); oliver.muensterer@med.uni-muenchen.de (O.M.);

**Keywords:** elbow fractures, fracture fixation, intramedullary, hospitals, university, learning curve, operative time, quality of life, retrospective studies

## Abstract

To assess changes in treatment modalities for supracondylar humerus fractures (SCHFs) at a large pediatric university hospital, we analyzed patient data from 2014 to 2022. A total of 233 SCHFs treated surgically at our hospital were included. To evaluate postoperative outcome and quality of life, DASH and EuroQol-5D-Y questionnaires were sent to patients. In addition to a significant fluctuation in fracture severity, we found an increase in training interventions (more surgeries were performed by trainees) and a significant decrease in surgery times after 2016. From 2020, there was a significant shift in the type of surgical method away from closed reduction with elastic stable intramedullary nailing (ESIN) and towards closed reduction and crossed K-wire osteosynthesis (CRK). Surgeries performed in the morning and evening hours increased, while those performed in the afternoon and after midnight decreased. After a mean follow-up of 4 years, there was no difference in elbow function between ESIN and open reduction and K-wires (ORK). Treatment with ESIN was equivalent to ORK in terms of function, at least in the medium-term follow-up. In summary, the combination of shifting treatment from SCHF to daytime hours, increasing trainee participation and using cross K-wire fixation instead of ESIN had no negative impact on surgery times. In our setting, these measures have reduced resource utilization and increased efficiency without compromising patient care.

## 1. Introduction

**Figure 1:**
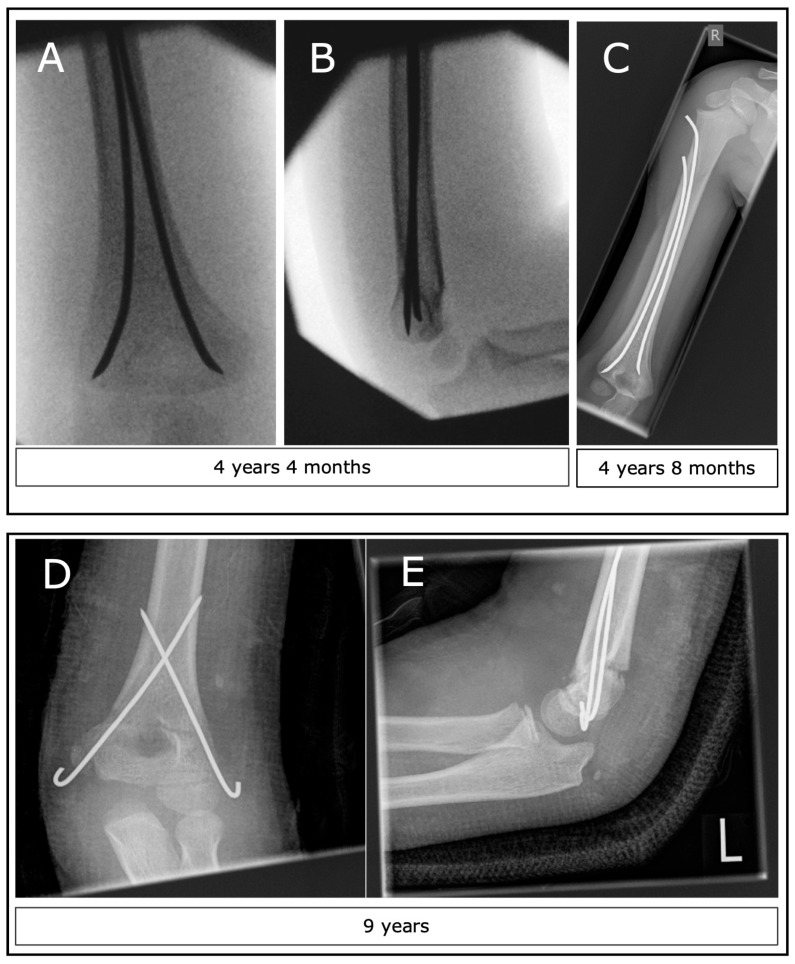
Two examples of surgical methods for SCHF. (**A**,**B**) Intraoperative images of a patient with a type II SCHF after closed reduction and ESIN. (**C**) The condition 4 months after surgery and before implant removal. (**D**,**E**) Images 1 week after open reduction and K-wire osteosynthesis plus plaster.

Supracondylar humerus fractures (SCHF) account for approximately 3 to 17% of pediatric fractures and are the most common type of elbow fracture in the pediatric population [1,2,3]. These fractures usually occur in children between the ages of 2 and 10 years, with a typical age range of 5 to 7 years. [4]. They predominantly affect the nondominant side and are evenly distributed among sexes [2,5]. Extension fractures comprise around 98% of these incidents, often arising from a fall onto an outstretched hand while the elbow is fully extended [2]. While type I fractures can be treated nonoperatively, the majority of displaced injuries (types II, III, and IV) necessitate surgical intervention [6]. There are several standard techniques for this fracture. Closed reduction and osteosynthesis with crossed K-wires (CRK) are often used. In some centers, closed reduction and elastic stable intramedullary nailing (ESIN) is offered as an alternative (Figure 1). The advantage of ESIN is that postoperative immobilization of the elbow with an upper arm cast is generally not required. Studies at our hospital have shown that ESIN is the preferred method for AO type II fractures. [7,8]. However, CRK remains the traditional surgical treatment for displaced SCHF that cannot be treated with conservative measures such as a simple plaster cast or the Blount’s sling technique, also known as the cuff and collar technique. [9,10]. For more severe fracture types, e.g., with soft tissue interposition, limited blood flow or compromise of sensory or motor function, surgical open reduction and osteosynthesis with K-wires (ORK) may be required [9,10,11].

In recent years, several new factors have emerged that influence the surgical treatment of pediatric fractures. Firstly, the COVID pandemic had a significant impact on the inpatient treatment of patients [12]. Secondly, due to the increasing nursing shortage in German hospitals, inpatient beds are scarce [13]. Third, the importance of structured teaching and training programs for surgical trainees has increased in the last decade [14]. Considering these contextual factors impacting the hospital environment as delineated above, our team endeavored to modify hospital procedures to enhance surgical training and relocate surgical interventions from nocturnal to diurnal hours, adhering to the ‘best time, best team’ principle. Concurrently, we transitioned our surgical technique from ESIN to a widespread and simplified approach employing CRK. Subsequently, we evaluated the effectiveness of these procedural changes in meeting our objectives of providing excellent patient care and outcomes. Our study, therefore, analyzed the frequency, preferred method, duration of surgery, time of day and percentage of teaching interventions in the treatment of SCHF at Dr. von Hauner Children’s Hospital, Ludwig-Maximilians-Universität München, a large university children’s hospital in a metropolitan area in Southern Germany over the last decade. Additionally, in a parallel questionnaire study, we scrutinized potential disparities in patient outcomes regarding the quality of life (QoL) and arm function between ESIN and K-wire osteosynthesis, which may warrant a reassessment of our long-term strategic approach.

## 2. Materials and Methods

### 2.1. Data Acquisition

We analyzed the DRG (Diagnosis Related Groups) data and the OPS (“Operation and Procedure Code”) data from 2014 to 2022 for all SCHF treated surgically at our center. The OPS system is the official German classification system for coding diagnoses, operations, procedures and medical interventions. We searched the system for the following OPS codes and DRGs: 5-790.23; 5-790.13; 5-794.13; 5-793.23; 5-790.16; S42.41; S42.42; S42.45. In addition, surgical reports and radiographs were used for analysis. All of the data in the files had been recorded prospectively by hospital staff according to our institution’s standard operating procedures. For retrospective analysis, these data were collected by A.B. and P.E. and assembled for further analysis. The focus was on the total number of operations performed annually and the fracture types according to the AO classification. While many centers use the Gartland classification, the AO classification is more widely used because of standardization and billing purposes [15,16]. The type of treatment technique (CRK, ESIN or ORK), operation times (OT, from the start of fracture reduction to the time of wound closure), time of day surgery started (TD) and the percentage of operations primarily performed by a resident in training assisted by a specialist (AS) were also recorded. “Specialist” was defined as a registered, certified pediatric surgeon, according to the German Medical Association (“Ärztekammer”).

We included all patients who were surgically treated for SCHF in our hospital in a preliminary analysis. We then excluded all patients who had a syndromal condition, a chronic neurological disorder or a cardiovascular problem that limited daily living activities. We also excluded patients with multiple fractures or an accidental brain injury.

### 2.2. Questionnaire Study

Questionnaires (DASH score—“disabilities of the arm, shoulder and hand” score as a measure of upper limb functionality) and the EuroQol-5D-Y (as a measure of quality of life) were sent to the 190 patients who had surgery for SCHF from 2013 to March 2020 (last known address recorded at last follow-up). Although the precedent data collection comprised the full years from 2013 to 2022, we included the year 2013 for this parallel investigation as our current computer system was established in mid-2013. Therefore, we were able to access the OPS data from that point onwards. For the questionnaire part of the study, we also included the additional patients we were able to retrieve data from in the year 2013. However, the questionnaires were sent right at the beginning of the COVID pandemic (March 2020) in order to exclude the impact of this potentially influential event and therefore analyzed quality of life before the year 2021. Both questionnaires are widely used and validated for childhood [17,18,19,20,21,22]. The data from the returned questionnaires were collected pseudonymously and the data were analyzed in terms of fracture type and technique used for the individual patient.

### 2.3. Data Analysis and Ethics Committee Approval

The retrospective analysis and anonymized storage of the data were performed using Excel. SPSS was used for statistical analysis. Student’s *t*-test was used to detect differences between groups unless otherwise stated in the text. *p*-values below 0.05 were considered statistically significant. The standard error of the mean (±SEM) was calculated as a measure of the range of variation and presented as error bars in the graphs unless otherwise described. The study was approved by the local ethics committee of the Ludwig-Maximilians-Universität München (ethics numbers 19-710 and 23-0139) and conducted according to the guidelines of the Declaration of Helsinki. Written informed consent was obtained from the patients and their legal guardians for the questionnaires, which was a prerequisite for further analysis.

## 3. Results

### 3.1. Patient Characteristics

From 2014 to 2022, a total of 253 supracondylar humerus fractures were treated surgically in our hospital. Of these, 233 were eligible for analysis as the data set was complete. In the long-term course, there was no significant change in the number of operated fractures per year for both fracture entities (see Supplement Figure S1).

The mean age at the time of injury was 6.2 years (min. 2; max. 17; standard deviation (SD) ± 2.5 years) and remained constant over the period. The mean proportion of male patients was 54.9%, with a higher fracture rate in boys (see Supplement Figure S2). The left side was affected in 64.2% of cases, and the right side in 35.8%.

### 3.2. Fracture Types

Between 2014 and 2022, we treated 27.4% type II fractures, 34.6% type III fractures and 38.0% type IV fractures according to the AO classification. Almost 40% of fractures in 2014 were type II fractures, decreasing to 12.5% by 2022. There were large differences in type IV fractures: In 2015, the proportion was almost two-thirds, whereas in 2017 and 2019, it was only a quarter (see Supplement Figure S3).

### 3.3. Time of Day (TD) the Surgery Started

**Figure 2:**
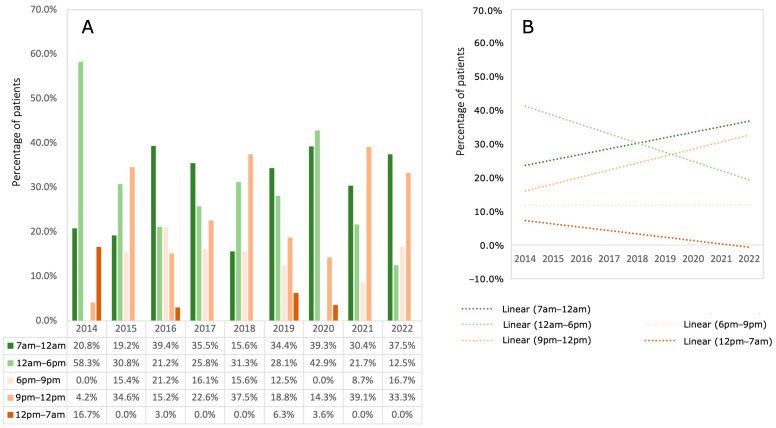
Time of the day on which the operations were performed. (**A**) The bars show the percentage of patients who underwent surgery at the highlighted time point. (**B**) The dotted lines show the corresponding linear regression of the percentages at each time point.

In terms of TD, most fractures were treated between 7 a.m. and 6 p.m. (79.1% in 2014, 52.1% in 2021 and 50.0% in 2022). On average, 27.7% (±8.0 SD) of fractures were treated after 9 p.m., with higher frequencies for the time window between 9 p.m. and 12 midnight. Treatments after 12 noon were a rarity, especially after 2014. An increase in fracture treatments was observed from morning until noon and between 9 p.m. and midnight, while treatments in the afternoon and after midnight decreased. The proportion of fractures treated in the early evening was constant during the observation period (Figure 2).

### 3.4. Teaching Interventions

**Figure 3:**
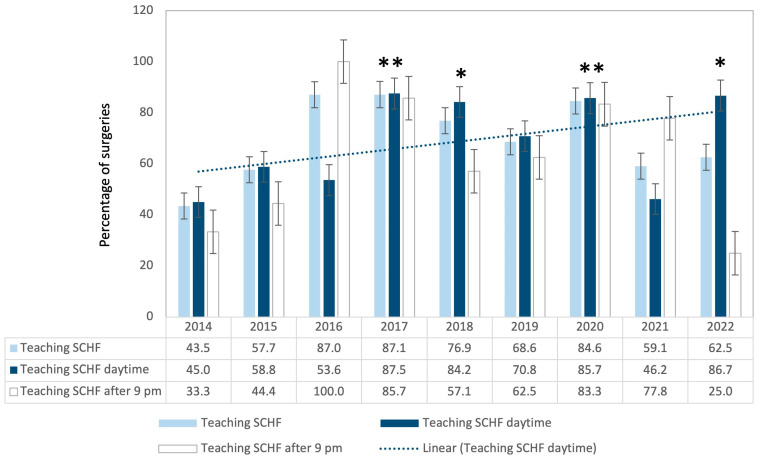
Percentage of surgeries in which the resident pediatric surgeon was assisted by a specialist (light blue: regardless of time of day; white: daytime (before 9 p.m.); dark blue: nighttime (after 9 p.m.); * *p* < 0.05 vs. 2014 daytime; ** *p* ≤ 0.01 vs. 2014 daytime); error bars show standard error of the mean (±SEM).

Between 2016 and 2017, there was a significant increase in the number of teaching procedures. While 43.5% of all surgeries were assisted in 2014, this share increased to 87.0% in 2016 and 2017 (see Figure 3).

### 3.5. Operative Time (OT)

**Figure 4:**
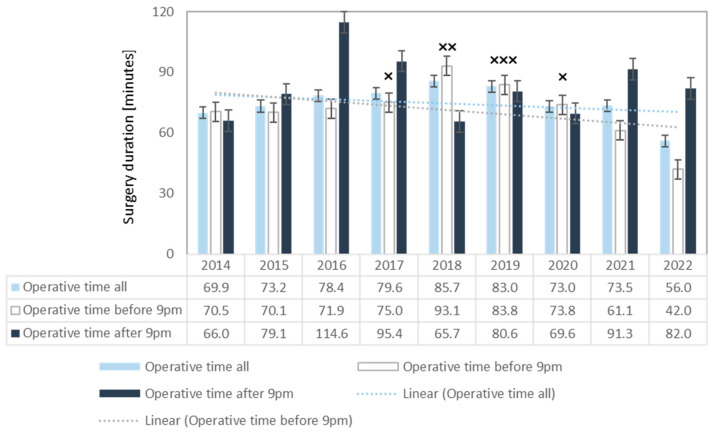
Development of operative time showing a decrease after 2018, especially for surgeries before 9 p.m. Depicted are the mean surgery durations ± standard error of the mean (±SEM); × indicate significant differences in operative time of surgeries before 9 p.m. compared with 2022; × *p* < 0.05; ×× *p* ≤ 0.01; ××× *p* ≤ 0.001.

After an initial increase in average OT (69.9 min in 2014 versus 85.7 min in 2017; *p* = 0.152), average surgery duration decreased to 56.0 min by 2022 (2018 versus 2022; *p* = 0.009). For surgeries performed after 9 p.m., there was no difference in operative times (66.0 min in 2014 versus 82.0 min in 2022, *p* = 0.269). From 2018, there was a continuous decrease in daytime OT (Figure 4).

### 3.6. Operative Technique (CRK, ESIN, ORK)

**Figure 5:**
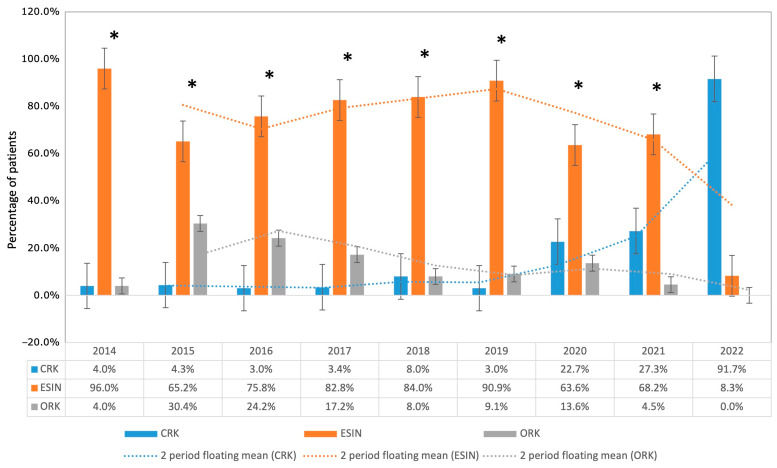
Percentage of patients who underwent surgery using different methods is shown. The data reflect a transition from ESIN to CRK, starting in 2020 and peaking in 2022. The error bars indicate ± standard error of the mean (±SEM), and the dashed lines represent the moving averages over two periods. * indicates a significant difference in the percentage of patients undergoing CRK or ESIN compared with 2022 (*p* ≤ 0.001).

While 96.0% were treated using ESIN in 2014, 91.7% were treated using CRK in 2022. Operative time remained statistically unchanged comparing 2014 with 2022 (*p* = 0.171). Both ESIN procedures in 2022 were type III fractures. The proportion of ORK did not differ between the years, even if there was a short peak in favor of ORK in 2015 and 2016 (see Figure 5).

### 3.7. Questionnaires

**Figure 6:**
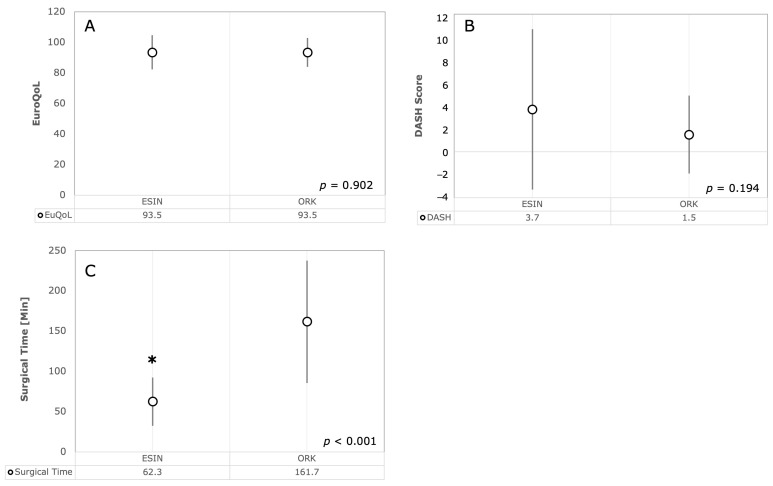
Results of questionnaires. (**A**) shows the results of EuroQoL and (**B**) the results of DASH scores of patients operated either via ESIN or ORK. Depicted are the mean scores ± standard deviation (StDev). (**C**) shows the mean operative time ± StDev for both procedures for the analyzed patients (n = 27 for ESIN and n = 21 for ORK). * *p*-values of *t*-tests < 0.05 were regarded as statistically significant.

Forty-nine patients responded to the questionnaires, resulting in a response rate of 25.8%. The average age at surgery was 6.7 years (minimum: 3 years; maximum: 11 years). In this group, there were 13 type II fractures, 12 type III fractures, and 23 type IV fractures. Among them, 27 patients received closed reduction and ESIN treatment, one patient received CRK, and 21 patients received ORK. Type III fractures were treated with ESIN in six cases and ORK in six cases. Type IV fractures were treated with ESIN in 13 cases and ORK in 10 cases. The average age at the time of questionnaire completion was 11.0 years (minimum 6 years, maximum 18 years). Therefore, the average follow-up time was 4.3 years. When comparing ESIN with ORK (Figure 6), neither the DASH score nor the total EuroQoL-5D-Y showed significant differences between the groups. However, the average operating time for ESIN was significantly shorter at 62 min, compared to ORK, which took 162 min (*p* < 0.001). In the subgroup analysis stratified by fracture types, no significant difference was observed between DASH and EuroQoL-5D-Y scores. However, significantly longer operation times were noted in Type 3 and 4 fractures for ORK. For Type 2 fractures, there was no statistically significant difference in operation time, most likely due to the fact that these patients were predominantly treated with ESIN.

## 4. Discussion

### 4.1. General Discussion

On the basis of national healthcare system changes and requirements, our team embarked on a strategic revision of hospital protocols, aiming to improve surgical training and transition surgical operations from night to day hours. This shift entailed transitioning from ESIN to CRK, a more universally adopted and streamlined method. We rigorously assessed the impact of these procedural modifications on achieving our goals, analyzing factors such as surgery frequency, preferred techniques, duration, timing, and the extent of educational interventions in SCHF treatment. Concurrently, a supplementary questionnaire-based study probed differences in patient outcomes, specifically in Quality of Life (QoL) and arm functionality. The questionnaire allowed us to compare the outcomes of ESIN and K-wire osteosynthesis. Our findings confirmed significant shifts in fracture management, including an increase in trainee-led surgeries and a concomitant, notable decrease in operation times after 2016. From 2020 onwards, a clear shift in surgical technique was observed, moving from ESIN to CRK, accompanied by a redistribution of surgery timings favoring morning and evening hours. The findings of our side study also suggest that the treatment efficacy of ESIN is comparable to that of ORK, at least in the context of medium-term outcomes.

Over the last decade, several factors have significantly influenced hospital care. Firstly, the COVID-19 pandemic has had a considerable impact on the availability of beds in pediatric hospitals. Moreover, as reported by Hoffmann et al., there has been a substantial shortage of beds due to a nursing deficit in recent years. This dearth of beds has been particularly pronounced within our metropolitan region [13]. In the investigated period we observed, the average annual number of SCHFs treated at our center was stable.

Regarding the time distribution of fracture treatments, the majority of fractures were managed during daytime in the earlier years, while towards the end of the observation period, a more equal distribution between daytime and nighttime surgery could be observed. Instances of treatment after 12 p.m. were infrequent, particularly after 2014. As expected, years without surgeries after midnight displayed higher percentages within the 9 p.m. to 12 a.m. time slot [23,24]. In summary, over the years, there was an increase in surgeries scheduled in the morning to noon and 9 p.m. to midnight treatments.

Although the former may be explained by a change in the urgency with which SCHFs should be operated upon due to an evolving paradigm shift, the latter may be associated with a greater number of severe fracture types observed in later years. In the past, Gartland Type III SCHF was considered an emergency procedure and required treatment within several hours of admission. However, starting in 2001, the timeframe was progressively extended due to new data in the literature [25,26,27]. Recently, Sullivan et al. demonstrated that there were no statistically significant differences for patients treated within or without an 18 h window regarding open or closed reduction, use of medial pins, or postoperative immobilization between cohorts, regardless of the severity of SCHF [28]. They also found no differences in postoperative complications, including nonunion, delayed union, stiffness, malunion, loss of reduction, iatrogenic nerve injury, or infection. This was true even when type II fractures were excluded. These new findings could have led to surgeons being more open to postponing surgery to the more suited surgical setting with higher resources during the next day, confirming the rule “best time, best surgeon, best team” [23,24]. However, in practice, the lack of operating room capacity on the following day may lead to pressure to treat an injury that is mainly suitable for elective surgery immediately as part of the emergency service in order to “make room” for upcoming and scheduled surgeries [24]. This is due to the fact that patients after SCHF repair can usually be discharged from the hospital on the first postoperative day.

The initial clinical examination of a patient is of utmost importance. The primary focus should not merely be on the neurovascular deficit, which manifests as an absent radial pulse and the phenomena termed the “pink hand” and “white hand syndrome”, but also on the rigorous evaluation of the soft tissue dynamics [29]. A hand that displays a pink and warm appearance is indicative of a limb that, despite being perfused, is devoid of a pulse [30]. Louahem and Cottalorda’s investigation unveiled 68 instances of acute ischemias and pink pulseless hands from a cohort of 404 SCHFs [30]. These underwent an emergent closed reduction in 63 patients to rejuvenate a well-perfused limb, warding off compartment syndrome. Conversely, a “white and cold hand” necessitated immediate surgical exploration and vascular rectification in five individuals. In a singular case, the development of compartment syndrome mandated supplementary intervention. The general consensus among the surgical community dictates that the “white and cold hand” necessitates immediate surgical exploration [31].

On the other hand, a fraction of surgeons advocate for a more conservative “watch and wait” approach after an immediate closed reduction in cases of “pink hand syndromes”, a strategy corroborated by some literature [31]. Nevertheless, findings from Stichhauer et al. revealed that out of their 125 pink pulseless hand cases post-SCHF, 9% necessitated vascular repair [32]. Concurrently, Delniotis et al., through a comprehensive systematic review, illustrated that post-closed reduction, the reappearance of a radial pulse in pink hands was observed in merely 30%. This underscores the advocacy for immediate surgical exploration if pulse restitution is not evident [31]. Our records include a case where venous interposition grafting was essential for brachial artery damage rectification. Therefore, this scenario, although a rarity, emphasizes the indispensability of vascular surgical backup, especially if the attending surgeon lacks expertise in such procedures.

It is notable that nerve afflictions are frequently concomitant with vascular deficits in SCHF [33]. Despite prevailing studies advocating for the predominant restoration of nerve functionality post-closed reduction in SCHF, recent research by Wilks et al. accentuates a swifter and more comprehensive recovery of median nerve afflictions post-open reduction and surgical exploration for the nerve. Thus, the optimal protocol for exclusive nerve deficits remains a topic of contention [33]. Our inclination leans towards early nerve exploration, particularly in the absence of discernible early recovery signs and if only closed reduction was performed. Of note, significant soft tissue edema invariably elevates the risk of compartment syndrome and often signifies the urgency of intervention, even devoid of a neurovascular compromise. This has to be taken into account at the time of initial examination and has to be reevaluated frequently if surgery is postponed.

In essence, the emergent surgical management of SCHF within the same diurnal cycle becomes imperative for injuries characterized by pronounced dislocation, jeopardized circulation, or sensory or motor function impairment. As a result, we have instituted the following criteria: Manifest or impending neurovascular deficits unequivocally indicate emergency surgery, warranting intervention even in the postmidnight hours. Rapidly evolving and pronounced soft tissue edema should be incorporated into the decision making. Recent systematic reviews, such as by Home et al., have spotlighted the ambiguities surrounding the term “normal perfusion”, emphasizing its inconsistent definition across multiple studies [29]. Consequently, the on-call surgeon always has the right and obligation to initiate instant intervention in doubtful cases and to seek advice from a more experienced colleague. Our residents always had an experienced surgeon specialist at their side since all operations were staffed by specialist surgeons. However, during the study period, we observed a significant shift from the specialist performing the operation with the trainee’s assistance to the trainee actually performing the surgery under the guidance of the specialist.

Consistent with the nature of a university hospital, the proportion of teaching procedures carried out by a resident under specialist supervision was high during the entire study duration. There was an overall rise in surgeries carried out with assistance, leading to an improvement in the training situation over the study period. Particularly looking at the daytime surgical teaching surgeries, which provide ample staff, they are best suited to teaching purposes. The variation observed over time might have been caused by the usual staff turnover in a university hospital. Consequently, there was a temporary upsurge in training requirements, as can be seen in the years 2016 and 2017. After a certain saturation of the distribution of surgical skills to the new staff, the number of teaching assistantships may decrease again. Interestingly, the third highest amount of training interventions was observed during the first year of the pandemic.

While there was an initial increase in the duration of the surgeries, the overall average operative time decreased by approximately 14 min. This can also be explained by a better distribution of surgical skills through intensified training. This fact disproves the general opinion that teaching in the operating room puts patients at risk due to prolonged anesthesia times. Weber et al. came to similar results in 2018, looking at operating time and outcomes after total knee replacement surgeries performed by surgical trainees under the supervision of senior surgeons [34].

Remarkably, there was a clear change in the type of surgery method. Starting in 2020, there was a significant increase in CRK and a simultaneous shift away from ESIN. CRK has a relatively steep learning curve, has low implant costs and, therefore, represents the most accepted method internationally. Also, there is no need for metal removal under general anesthesia in CRK. Usually, the wires can then be removed after 4–5 weeks during an outpatient visit. Migration of pins with the need for surgical removal is a rare event [35]. As Schneidmüller et al. demonstrated in 2022, there is no difference between percutaneous K-wires and subcutaneous K-wires in terms of pain and anxiety experienced by children and parents [36]. The patients, as well as the families, are just as afraid of anesthesia as they are of the pull of a K-wire extending through the skin. Percutaneous K-wires spare the children a second procedure, eliminate the risk posed by general anesthesia, reduce costs, free up hospital beds and enable parents to go to work. Accordingly, we switched from subcutaneous to percutaneous intraoperative K-wire placement and removal in the outpatient clinic.

Surgeons in favor of ESIN claim the advantage of not requiring postoperative immobilization [8]. The children are allowed to move the elbow freely and usually show a fast mobilization of the upper extremity with good rotational control and good functional outcome [37]. Nevertheless, ESIN is technically more demanding, yields longer surgeries and has higher implant costs. For the removal of the ESIN—which is due after 3–6 months—general anesthesia and a minor surgery are definitely necessary. The change in the surgical method at our institution may have several reasons. First, the ESIN is well suited to address type II fractures, as already shown by Lacher et al. in 2012 [7]. As shown in 2021 and 2022, there was an increased incidence of more severe type III and IV fractures, which often leads to surgeons resorting to the simpler K-wire technique to achieve a good result. We have instituted a protocol whereby, following a closed reduction attempt lasting a maximum of 15 min, we transition to an open approach. The primary rationale behind this protocol is to prevent undue prolongation of the surgical procedure and to minimize potential trauma to the fracture ends, which might result in a critically unstable situation that is even more challenging to manage. Fortunately, we found no ORK procedure in 2022. Consequently, we attribute the observed decrease in open reductions (notably in 2022) to enhanced training methodologies. Of note, we solely perform surgeries for SCHF in the supine position. An arm table is applied depending on the choice of the surgeon. Sapienza et al. investigated the influence of the positioning of the patient in a recent systematic review and found no differences regarding the functional and radiographic outcome of SCHF [38].

Eberl et al. showed a decreased rate of iatrogenic ulnar nerve injury when performing ESIN compared to CRK (0.4% vs. 15%) [39]. However, Liu et al. showed superior mechanical stability for CRK compared to ESIN or external fixation [40]. Greve et al. also showed slightly superior results for CRK compared to ESIN regarding intraoperative fluoroscopy time and duration of surgery, although the nature of the study was retrospective and a cast was routinely applied also in the ESIN group [41]. We shifted from ESIN’s to K-wires because of our impression that we had better postoperative reduction with K-wires. In fact, as seen in Figure 1, our analysis confirmed our suspicion retrospectively. Of note, we do not perform a miniulnar incision in order to preserve the ulnar nerve of an iatrogenic injury [42]. In 2020, a US-trained pediatric surgeon took over as head of the department. Since CRK is widely recommended in the US as the treatment of choice for SCHF AO types II-III [18], Therefore, we believe that this last point gave the final impact—besides the named scientific arguments—which led to the significant change.

In our parallel questionnaire study, after an average examination period of 4.3 years, we found no significant difference in terms of functionality and quality of life of the children when comparing ORK with ESIN. This statement was confirmed by the standardized DASH score and the EuroQoL score. However, we studied only patients who underwent surgery before the COVID-19 pandemic to avoid possible bias in the interpretation of the results [43,44]. Despite significant differences with regard to operative time, there was an equivalent result for ESIN and ORK, at least in the mid-term. Therefore, our data indicate that shorter postoperative examination intervals may be required to confirm this advantage. Of note, a shorter recovery time and return to sport can be essential for the development of a child. Nevertheless, to date, there is no evidence that the ESIN method is favorable compared to CRK as randomized controlled trials are still missing.

### 4.2. Study Limitations

There are several factors that limit our analysis. While the data were collected prospectively, the analysis is retrospective in nature. The average follow-up time of the questionnaires was only 4.3 years, so only medium-term results are available. In addition, we only examined the clinical outcomes of patients who underwent surgery before the COVID pandemic to avoid potential bias in the interpretation of the results. There are, therefore, no short follow-up observations that could show the advantages or disadvantages of one of the methods in the immediate temporal proximity to the intervention. It must also be taken into account that either the ESIN or the ORK method was used in the period under consideration. Therefore, no data are available that show a difference between ESIN and CRK, as well as CRK and ORK. The low response rate of less than 26% is probably due to the fact that families with young children in a large city move more frequently than families in smaller regions, and we were unable to determine the correct current address at the time the questionnaires were sent to the families.

## 5. Conclusions

In conclusion, our comprehensive evaluation led to transitioning to predominantly diurnal management of SCHF, enhanced with increased trainee involvement and the adoption of the cross-K-wire fixation over ESINs. Remarkably, there has been a noticeable decline in the frequency of late-night surgical interventions. Surgical training has seen significant improvements, and simultaneously, the mean duration of surgical procedures has diminished, pointing to increased efficiency. Notably, the streamlining of the surgical method has simplified the overall process. Within our institutional framework, these multifaceted strategies have not only optimized resource allocation and bolstered operational efficiency but have also done so without any compromise to the gold standard of patient care. We thus advocate for the adoption of the following methodologies: Firstly, the enhancement and optimization of surgical education to ensure a more widespread dissemination of expertise; secondly, the strategic realignment of procedures to daylight hours, which not only facilitates superior surgical training but also adheres to the principle of ‘optimal time, optimal surgical setting’; and thirdly, the simplification of surgical techniques. These measures are propounded with the primary objective of achieving the best possible patient outcomes.

## Data Availability

The data presented in this study are available on request from the corresponding author. The data are not publicly available due to privacy reasons.

## Supplementary Materials

The following supporting information can be downloaded at: https://www.mdpi.com/article/10.3390/children11010027/s1, Figure S1. Number of patients operated on per year for SCHF; Figure S2. Annual distribution of male and female patients undergoing surgery for SCHF; Figure S3. Annual distribution of fracture types according to the AO classification; Figure S4. The results of the DASH, EuroQoL-5D-Y questionnaires and surgery time; Table S1. The results of the DASH, EuroQoL-5D-Y questionnaires and surgery time; Table S2. The results of statistical analysis of the DASH, EuroQoL-5D-Y questionnaires and surgery time.
