# Supplementary material for: Intrainstitutional Changes of the Treatment of Supracondylar Humerus Fracture in Children over a Period of 9 Years
Source: Children (Basel). 2023 Dec 26;11(1):27. doi: 10.3390/children11010027 (PMC10814588; doi:10.3390/children11010027)
Supplement: Supplementary file 1 [file children-11-00027-s001.zip › children-2753829-supplementary.pdf]

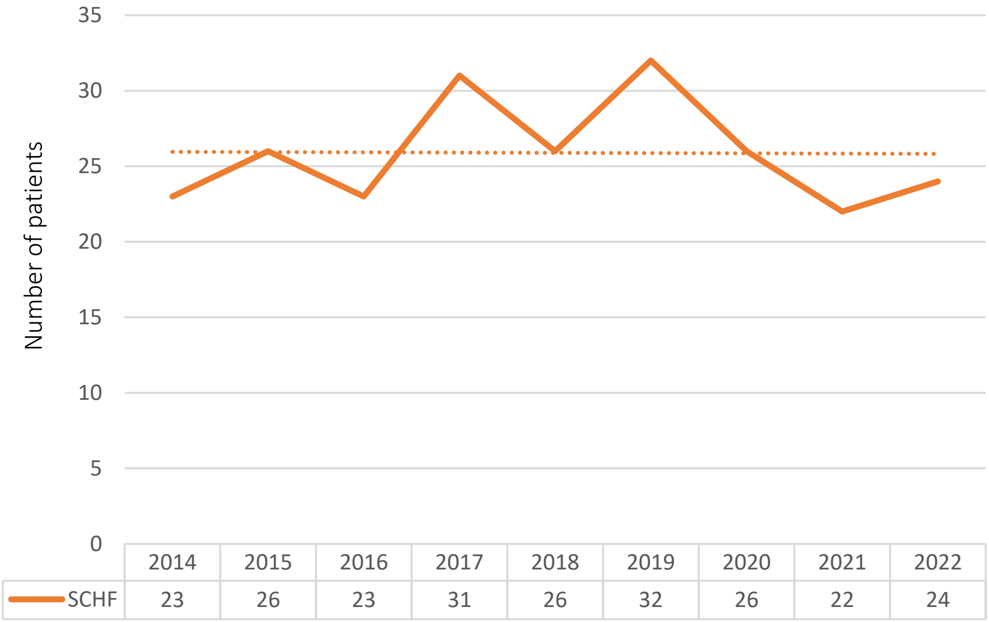

Figure S1. Number of patients operated on per year for SCHF. The dashed line shows the average of 25.9 fractures per year.

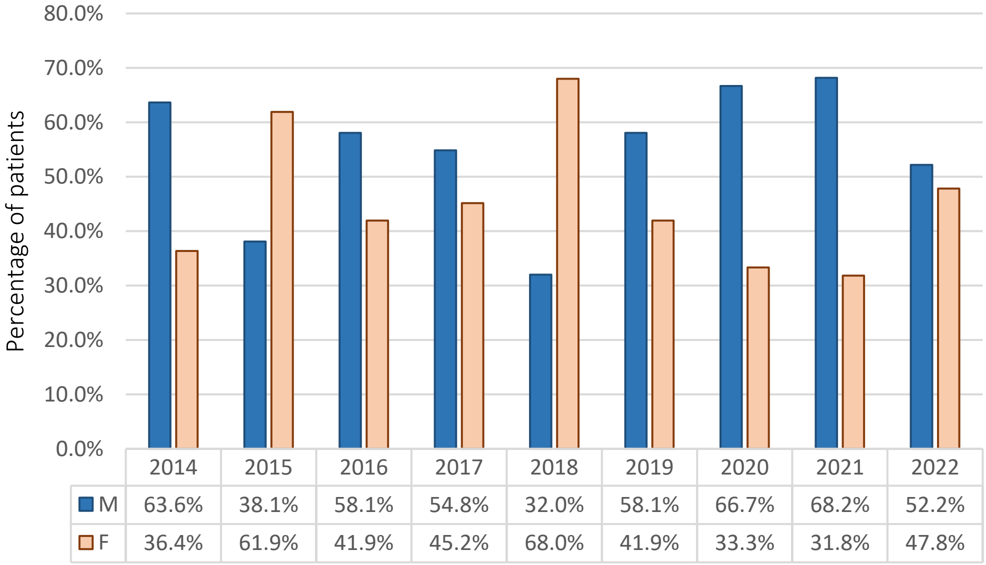

Figure S2. Annual distribution of male and female patients undergoing surgery for SCHF. M-Males, F-Females.

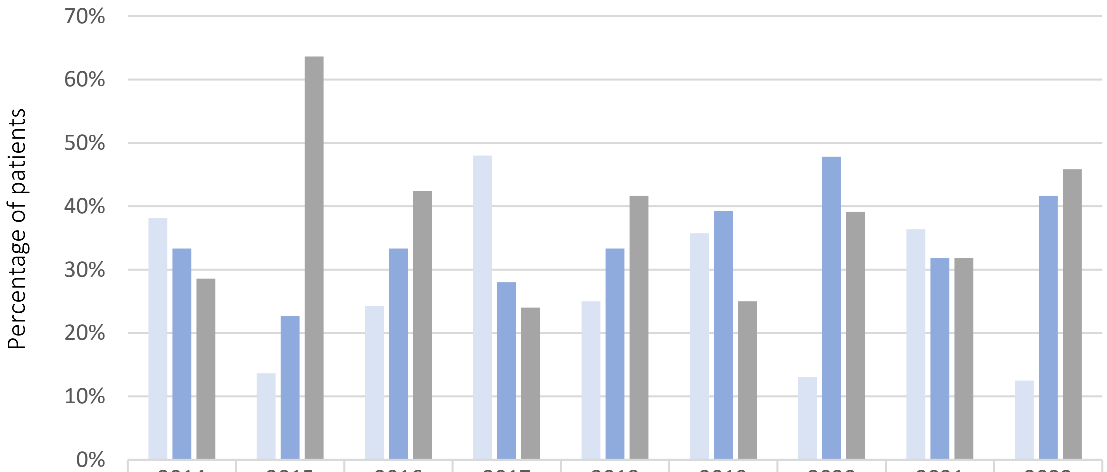

	2014	2015	2016	2017	2018	2019	2020	2021	2022
Type II	38.1%	13.6%	24.2%	48.0%	25.0%	35.7%	13.0%	36.4%	12.5%
Type III	33.3%	22.7%	33.3%	28.0%	33.3%	39.3%	47.8%	31.8%	41.7%
Type IV	28.6%	63.6%	42.4%	24.0%	41.7%	25.0%	39.1%	31.8%	45.8%

Figure S3. Annual distribution of fracture types according to the AO classification. The bars show the percentage age of the patients grouped according to the type of fracture.

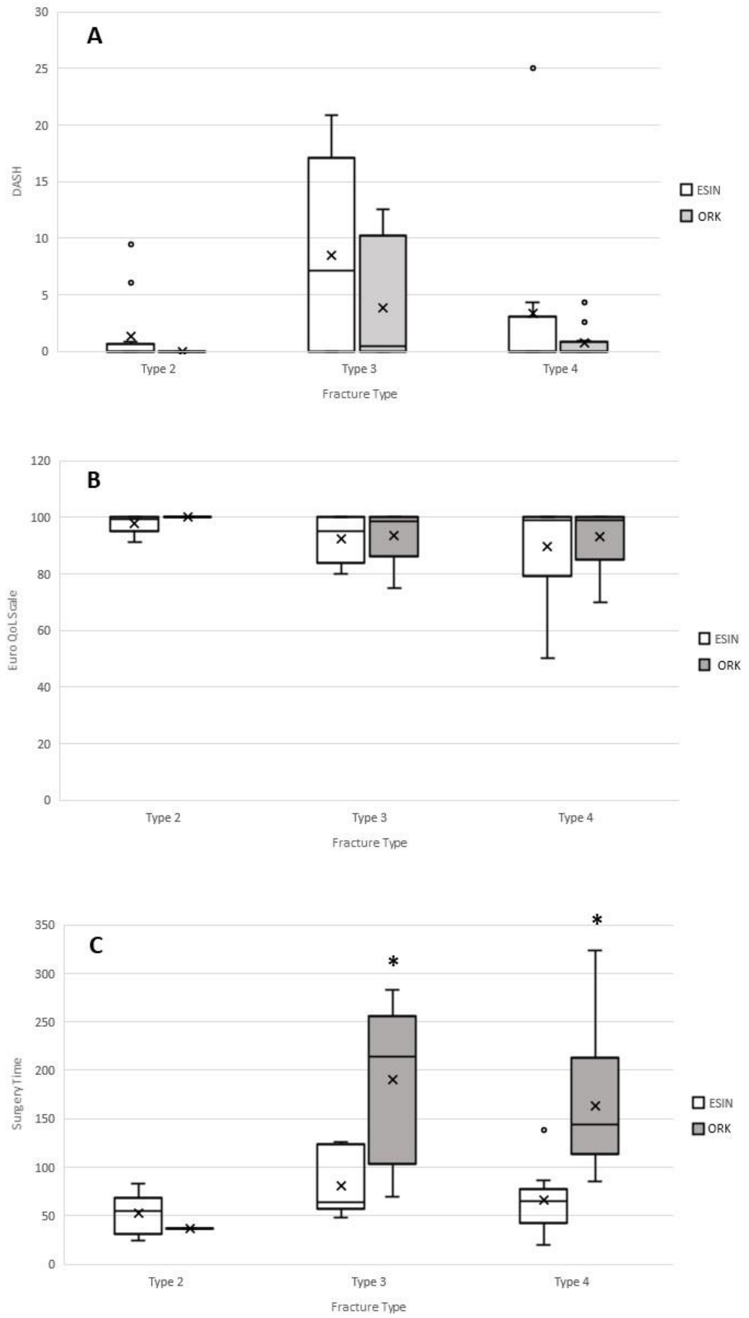

Figure S4. The results of the DASH, EuroQoL-5D-Y questionnaires and operative time (Surgery time) are presented and were categorized by fracture type and surgical method. As only one patient was treated using the CRK method, this case was excluded from further analysis. Results are presented by boxplots: bottom and top of box = 25th and 75th percentiles, X = mean, thin line inside box = median, I bar = maximum and minimum of data, and circle = outlier. * $p \leq 0.05$; ** $p \leq 0.001$.

Fracture Type			N	Mean	Standard deviation ±	Standard error of the mean ±
Type 2	DASH	ESIN	12	1.4	3.1	0.9
		CRK	1	0.0		
	EuroQoL-5D-Y	ESIN	12	97.8	3.0	0.9
		CRK	1	100.0		
	Surgery Time	ESIN	12	52.2	20.4	5.9
		CRK	1	37.0		
Type 3	DASH	ESIN	6	8.5	9.6	3.9
		ORK	6	3.8	5.7	2.3
	EuroQoL-5D-Y	ESIN	6	92.5	8.8	3.6
		ORK	6	93.7	9.9	4.1
	Surgery Time	ESIN	6	81.0	34.2	14.0
		ORK	6	190.3	81.5	33.3
Type 4	DASH	ESIN	10	3.4	7.7	2.5
		ORK	13	0.7	1.3	0.4
	EuroQoL-5D-Y	ESIN	10	89.6	16.4	5.2
		ORK	13	93.0	10.1	2.8
	Surgery Time	ESIN	10	65.7	33.5	10.6
		ORK	13	163.8	68.6	19.0

Table S1. The results of the DASH, EuroQoL-5D-Y questionnaires and surgery time categorized by fracture type and surgical method.

Fracture Type			t-Test for mean values			
			Significance		95% Confidence intervall of the difference	
			1-tailed p	2-talied p	Lower value	Upper value
Type 2	DASH		0.340	0.679	-5.703	8.433
	EuroQoLScale		0.244	0.489	-9.166	4.666
	SurgeryTime		0.245	0.489	-31.495	61.828
Type 3	DASH		0.163	0.326	-5.412	14.782
	EuroQoLScale		0.417	0.834	-13.240	10.907
	SurgeryTime		0.006*	0.013*	-189.787	-28.880
Type 4	DASH		0.120	0.239	-1.888	7.153
	EuroQoLScale		0.273	0.546	-14.923	8.123
	SurgeryTime		<0.001**	<0.001**	-147.336	-48.802

Table S2. The results of statistical analysis of the DASH, EuroQoL-5D-Y questionnaires and surgery time categorized by fracture type. P-values were calculated analysing the difference between the surgical methods (ESIN vs. ORK for Type 3 and 4; ESIN vs CRK for type 2).
